# CircTP63 promotes cell proliferation and invasion by regulating EZH2 via sponging miR-217 in gallbladder cancer

**DOI:** 10.1186/s12935-021-02316-w

**Published:** 2021-11-17

**Authors:** Shouhua Wang, Huanjun Tong, Tingting Su, Di Zhou, Weibin Shi, Zhaohui Tang, Zhiwei Quan

**Affiliations:** 1 Department of General Surgery, Xinhua Hospital, Shanghai Jiao Tong University School of Medicine, Shanghai, 200092 China; 2 Shanghai Key Laboratory of Biliary Tract Disease Research, 1665 Kongjiang Road, Shanghai, 200092 China; 3 Department of Blood Transfusion, Xinhua Hospital, Shanghai Jiao Tong University School of Medicine, Shanghai, 200092 China; 4 Department of General Surgery, Xinhua Hospital, Shanghai Jiao Tong University School of Medicine, No.1665 Kongjiang Road, Yangpu District, Shanghai, 200000 China

## Abstract

**Background:**

Gallbladder cancer (GBC) is the most common biliary tract malignancy and has a poor prognosis in patients with GBC. CircRNA TP63 (circTP63) has been implicated in cell proliferation and invasion in some tumor progress. The study aims to investigate the clinical significance and functional role of circTP63 expression in GBC.

**Methods:**

The expression of circTP63 in GBC tissues or cells was detected by qRT-PCR and the association between circTP63 expression and prognosis of GBC patients was analyzed. CCK8 assay, flow cytometry analysis, transwell assay and in vivo studies were used to evaluate the cell proliferation and invasion abilities after circTP63 knockdown in GBC cells. Luciferase reporter assays and RNA pull-down assay were used to determine the correlation between circTP63 and miR-217 expression. Besides, western blot analysis was also performed.

**Results:**

In the present study, we showed that circTP63 expression was upregulated in GBC tissues and cells. Higher circTP63 expression was associated with lymph node metastasis and short overall survival (OS) in patients with GBC. In vitro, knockdown of circTP63 significantly inhibited cell proliferation, cell cycle progression, migration and invasion abilities in GBC. Besides, we demonstrated that knockdown of circTP63 inhibited GBC cells Epithelial-Mesenchymal Transition (EMT) process. In vivo, knockdown of circTP63 inhibited tumor growth in GBC. Mechanistically, we demonstrated that circTP63 competitively bind to miR-217 and promoted EZH2 expression and finally facilitated tumor progression.

**Conclusions:**

Our findings demonstrated that circTP63 sponged to miR-217 and regulated EZH2 expression and finally facilitated tumor progression in GBC. Thus, targeting circTP63 may be a therapeutic strategy for the treatment of GBC.

## Introduction

Gallbladder cancer (GBC) is one of the most common digestive tract tumors worldwide [1]. GBC is characteristically diagnosed at advanced stages due to the absence of specific signs and symptoms, and only a small population of GBC patients is suitable for the surgical resection [2, 3]. Most gallbladder cancer patients have an extremely poor prognosis, and the 5-year overall survival rate is less than 5% [4]. The non-surgical therapies for GBC patients are primarily composed of chemotherapy, radiotherapy, targeted therapy. Recently, studies have found that molecular targeted therapeutics including fibroblast growth factor receptor (FGFR), MEK, ERBB2 or PI3-Kinase inhibitors have been explored, which provide hope for gallbladder cancer treatment [5]. Therefore, elucidating the mechanism of the occurrence and development and validating existing novel molecular target to improve therapeutic effects of GBC patients are needed.

Circular RNAs (circRNAs) are a type of endogenous non-coding RNA that do not have a 5′-cap or a 3′-polyA tail and are implicated in a variety of biological functions [6, 7]. Recently, RNA-sequences revealed that circRNAs are involved in diagnosis, prognosis, development, and drug resistance in some tumors. CircRNAs were found to regulate cell apoptosis, cell proliferation, cell migration and tumor metastasis by regulating gene expression [8]. Such as, hsa_circRNA_100269 expression is downregulated in gastric cancer and upregulated hsa_circRNA_100269 inhibits cell growth and tumor metastasis through inactivating PI3K/Akt axis [9]. Up-regulated circBACH2 is found in triple-negative breast cancer and contributes to cell proliferation, invasion, and migration of triple-negative breast cancer [10]. circCCDC66 expression is upregulated in thyroid cancer and promotes cell proliferation, migratory and invasive abilities and glycolysis through the miR-211-5p/PDK4 axis [11]. Hsa_circ_0068515, designated as circTP63, is reported in lung squamous cell carcinoma (LUSC) and correlates with larger tumor size and higher TNM stage in LUSC patients. Besides, upregulated circTP63 is also identified to promote cell proliferation by functioning as a ceRNA to upregulate FOXM1 in LUSC [12]. Another study shows that circular RNA circTP63 enhances estrogen receptor-positive breast cancer progression and malignant behaviors through the miR-873-3p/FOXM1 axis [13]. In hepatocellular carcinoma (HCC), circTP63 is significantly upregulated in HCC tissues and cell lines, and circTP63 overexpression promotes tumor progression by sponging miR-155-5p and upregulating ZBTB18 expression [14]. However, its role in GBC progression remains unknown.

In the study, we firstly demonstrated that circTP63 expression was significantly upregulated in GBC tissues and cells. Upregulated circTP63 expression notably associated with short survival rate of GBC patients. Functionally, knockdown of circTP63 inhibited cell proliferation, cell cycle progression, and cell invasion abilities in GBC and suppressed tumor growth in vivo. Besides, we demonstrated that knockdown of circTP63 inhibited cell EMT process in GBC. Mechanistically, we showed that circTP63 could competitively bind to miR-217 and promoted level of EZH2 expression, which finally facilitated tumor progression. Thus, these results provided better understand for the regulatory role of circRNAs in GBC progression, and could improve the diagnosis and therapies of GBC.

## Materials and methods

### Patients tissue samples

The study was performed in accordance with the Declaration of Helsinki and the guidelines of the committee of the Human Ethics Committee of Xinhua Hospital. A total of 39 snap-frozen GBC tissues and paired adjacent normal tissues were acquired from patients diagnosed with GBC at Xinhua Hospital between March 2009 and March 2016. All the enrolled patients of this study had never received preoperative therapy and tissue samples were collected and frozen in liquid nitrogen immediately after surgical resection. The information for GBC patients was shown in Table 1. All of participants signed informed consent before this study.

**Table 1:** The association between circTP63 expression and clinicopathological factors in GBC patients

Clinicopathological factors	The number of patients (n = 39)	CircTP63 expression		P value
		Lower (n = 19)	Higher (n = 20)	
Age				0.839
≤ 60	24	12	12	
> 60	15	7	8	
Gender				0.798
Male	11	5	6	
Female	28	14	14	
Tumor size				0.894
< 5 cm	16	8	8	
≥ 5 cm	23	11	12	
Histological grade				0.429
Well and moderately	21	9	12	
Poorly and others	18	10	8	
Lymph node metastasis				0.038*
Negative	18	12	6	
Positive	21	7	14	
Adjacent organ invasion				0.433
No	16	9	7	
Yes	23	10	13	
TNM stage				0.264
I–II	19	11	8	
III–IV	20	8	12	

*TNM* tumor-node-metastasis

^*^P  <  0.05

### Cell lines culture

Three human GBC cell lines (NOZ, GBC-SD, and SGC-996) and the normal human intrahepatic biliary epithelial cell line HIBEC used in the present study were purchased from Cell Bank of the Chinese Academy of Science (Shanghai, China). Cells were cultured in DMEM (Gibco, Invitrogen, USA) supplemented with 10% fetal bovine serum (FBS) (Gibco, Invitrogen, US) and 0.5% penicillin/streptomycin (Gibco, Invitrogen, USA). Cells were maintained at 37 °C in a humidified atmosphere containing 5% CO_2_.

### Cell transfection

Cell transfection was performed with Lipofectamine 2000 or 3000 Reagent (Invitrogen, CA, USA) according to the manufacturer’s protocol. The two siRNA against circTP63 and the si-negative control (si-NC) oligos were purchased from Gene Pharma (Shanghai, China). The sequences for si-circTP63-1, si-circTP63-2 or si-NC were as follows: si-circTP63-1,5′-GCCAACAGUGAGGGGCCGU-3′; si-circTP63-2,5′-CAACAGUGAGGGGCCGUGAGA-3′; si-NC, 5′-UUCUCCGAACGUGUCACGU-3′. MiR-217 mimic, miR-217 inhibitor and miR-NC were obtained from Gene Pharma (Shanghai, China).

### CCK8 assays

NOZ and SGC-996 cells were seeded in 96-well plates (3 × 10^3^ cells per well). After cells were transfected at 1–5 days, cells were added with 10 μl of the CCK-8 solution (Dojindo Laboratories, Kumamoto, Japan) in each well of the plate. Then, cells were incubated for 2 h in the incubator. Finally, the absorbance was detected at 450 nm using a microplate reader (BioTek Instruments, Inc., Winooski, VT, USA).

### Flow cytometry analysis

Transfected cells were harvested, washed and then were fixed with 70% ethanol at − 20 °C overnight, Next, after RNase digestion, the cells were stained with 20 μg/ml Propidium iodide (PI; Beyotime, Shanghai, China) at 37 °C for 30 min, and 100 μg/ml RNase A was subsequently added to the cells and incubated in a 4 °C dark room for 30 min. Cell cycle was examined by flow cytometry using the FACS Calibur system (BD Biosciences, San Jose, CA, USA).

### Cell migration and invasion assays

Cell migration or invasion assays were performed by transwell plates (BD Falcon, USA) and were coated without or with Matrigel in 24-well transwell chambers with 8 mm pore polycarbonate filters (Millipore, Billerica, MA, USA). 1 × 10^5^ cells were cultured on the upper chamber in medium without serum, while the lower chamber was added with 10% fetal bovine serum (FBS) (Gibco, Invitrogen, USA). After transfection at 48 h, cells on the lower layer of the membrane were stained using 1% crystal violet for 30 min at room temperature. The cell number was counted by using an Olympus microscope and five fields were randomly selected to count the cells (Magnification 200× or Magnification 100×). All assays were independently performed in triplicate.

### Quantitative real-time PCR (QRT-PCR) analysis

Total RNA was extracted from tissues or cells using TRIzol reagent (Takara, Japan). The cDNA was synthesized using a Prime Script RT reagent kit (Takara, Japan). The mRNA expression was analyzed by using SYBR Green Real-Time PCR Master Mixes (Thermo Fisher Scientific, USA) by an ABI 7900 Fast Thermal Cycler (Applied Biosystems; Thermo Fisher Scientific, USA). The GAPDH or U6 was used as reference. The primer sequences were as follow: the circTP63 forward: 5′-GCCCTCACTCCTACAACCATT-3′; circTP63reverse: 5′-TTGTGTGCTGAGGAAGGTACT-3′; TheEZH2-forward: 5′-TGCAGTTGCTTCAGTACCCATAAT-3′; EZH2-reverse: 5′-ATCCCCGTGTACTTTCCCATCATAAT-3′; GAPDH-forward: 5′-AAGGTGAAGGTCGGAGTCA-3′; GAPDH-reverse: 5′-GGAAGATGGTGATGGGATTT-3′; U6-forward: 5′-CTCGCTTCGGCAGCACA-3′; U6-reverse: 5′-AACGCTTCACGAATTTGCGT-3′. The relative mRNA expression was calculated using the 2^−ΔΔCt^ methods.

### Nuclear-cytoplasmic fractionation

Cytoplasmic and nuclear RNAs were isolated using NE-PER Nuclear and Cytoplasmic Extraction Reagents (Thermo Scientific, USA) following all manufacturer protocols. We followed this experiment with qRT-PCR analysis and GAPDH and U6 were used as controls.

### Western blotting assays

Total protein was extracted using a RIPA buffer (Beyotime, Beijing, China). An equal amount of total protein was separated on SDS-polyacrylamide gel electrophoresis (SDS-PAGE) and then transferred onto PVDF membranes (Millipore, Billerica, MA. USA). The membrane was blocked with 5% non-fat milk and incubated with the primary antibody with E-cadherin (1:1000, Cell Signaling Technology, Houston, TX, USA). Vimentin (1:1000, Cell Signaling Technology, Houston, TX, USA), EZH2 (1:1000, Cell Signaling Technology, Houston, TX, USA) and GAPDH (1:2000, Abcam) overnight at 4 °C. Next, the secondary antibodies were added for 1.5 h and then each protein band was detected by the ECL detection system (Amersham Biosciences, Buckinghamshire, UK).

### Luciferase reporter assays

The wide type (WT) circTP63 or EZH2 3′-untranslated region (UTR) containing miR-217 targeting sequence and the mutated type (MUT) was amplified and cloned into the luciferase reporter plasmid psicheck-2 vector (Promega, Madison, WI). Cells were collected and lysed for luciferase detection 48 h after transfection. Luciferase activities were measured using the Dual-Luciferase Reporter Assay System (Promega, USA). The relative luciferase activity was normalized against to the Renilla luciferase activity.

### Biotin-coupled RNA pull down

The 3′end biotinylated miR-217 or control RNA was designed and synthesized by GenePharm (Shanghai, China). NOZ were transfected with 50 nM of biotin-labeled miRNAs. Streptavidin-coupled Dynabeads (Invitrogen) were washed, resuspended in the buffer and then was added with the biotin-labeled miRNAs. After incubating at room temperature for 10 min, the coated beads were separated with a magnet for 2 min. The pulled-down RNA was extracted by Trizol reagent and followed by qRT-PCR analysis.

### In vivo* xenograft experiments*

Xenograft experiments (n  = 5/per group) or metastatic experiments were performed by using 3-week BALB/c nude mice. All animal protocols were approved by the Institutional Animal Care and Use Committee at Xinhua Hospital. 1 × 10^5^ NOZ cells were transfected and were subcutaneously injected into the flank. The tumor volume and weight were evaluated every week, Tumor volume (mm^3^)  =  (length)  ×  (width)^2^/2. After 4 weeks, mice were sacrificed and tissues processed for further histological analysis. According to the AVMA Guidelines for the Euthanasia of Animals, all the mice were euthanized with an intraperitoneal injection of a three-fold dose of barbiturates. After that we removed tumors immediately and measured the length, width and weight of the tumors. No mice died accidentally during feeding.

### Immunohistochemistry (IHC)

Immunohistochemistry was performed using tumor issues by HRP-conjugated secondary antibody for staining and DAB Kit (ZSGB-BIO, China) for color development. The antibodies used for immunochemistry staining were below: E-cadherin (Cell Signaling Technology, Houston, TX, USA), Vimentin (Cell Signaling Technology, Houston, TX, USA) and Ki-67(Cell Signaling Technology, Houston, TX, USA). The slides were scored based by the intensity of the staining and the percentage of cells stained. Slides were visualized at  ×  200 magnification for scoring the staining intensity: no color for 0 points; light yellow for 1 point; yellow for 2 points; brown for 3 points. For each slide, 5 high magnification (× 400) field were randomly selected to count positive cells ratio: less than 10% for 0 point; 10% to 25% for 1 point; 25% to 50% for 2 points; 50%-100% for 3 points. The 2 scores were added up as final score.

### Statistical analysis

All statistical analyses were performed using GraphPad Prism (GraphPad Software, Inc. La Jolla, USA). The data were presented as mean  ±  SD, and compared by Student’s *t *test or ANOVA with Tukey test. A P value of less than 0.05 was considered statistically significant.

## Results

### CircTP63 expression is upregulated in GBC and correlates with poor prognosis

**Fig. 1:**
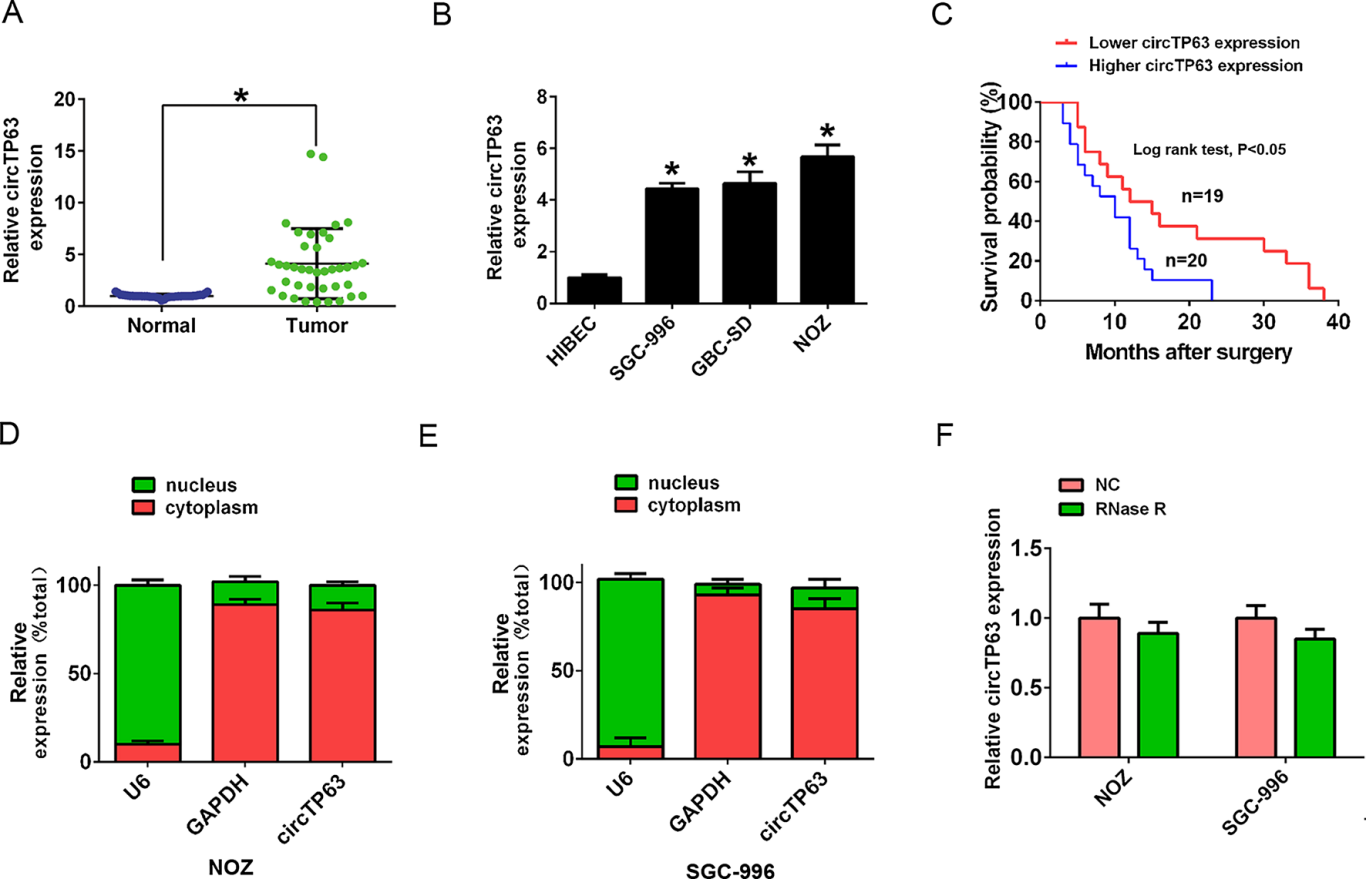
CircTP63 expression is upregulated in GBC. **A** The relative expression of circTP63 was detected in GBC tissues and adjacent normal tissues by qRT-PCR analysis. **B** The relative expression of circTP63 was detected in three GBC cell lines (NOZ, SGC-996 and GBC-SD) and the human intrahepatic biliary epithelial cell line HIBEC by qRT-PCR analysis. **C** Kaplan–Meier survival curves indicating the correlation between circTP63 and overall survival (OS) in GBC patients. **D**, **E** Nucleocytoplasmic fractionation experiment demonstrated that circTP63 expression was mainly distributed in the cytoplasm. **F** QRT-PCR was performed to detect the circTP63 expression from control or digested RNAs using RNase R exonuclease in NOZ or SGC-996 cells. Data are shown as mean  ±  SD, *P  < 0.05

To explore the clinical significance of circTP63 expression in GBC, we detected the circTP63 expression collected from 39 pairs of GBC tissues and adjacent normal tissues by qRT-PCR. The results showed that circTP63 expression was dramatically upregulated compared with adjacent normal tissues in patients with GBC (Fig. 1A). Moreover, we also detected that circTP63 expression was higher in three GBC cell lines than in HIBEC cells (Fig. 1B). The 39 cases of patients were divided into circTP63 higher expression and lower expression groups according to the median expression value. The association between circTP63 expression and clinicopathological characteristics was analyzed. The results displayed that circTP63 expression was associated with lymph node metastasis (Table 1, P  <  0.05). Kaplan–Meier analyses and log rank test showed that higher circTP63 expression showed a poor overall survival rate in GBC patients compared with lower circTP63 expression (Fig. 1C). We next examined the subcellular localization of circTP63, and the results demonstrated that circTP63 expression was predominately distributed in the cytoplasm than that in the nucleus in NOZ and SGC-996 cells (Fig. 1D, E). Total RNA isolated from NOZ and SGC-996 cells was exposed to RNase R and the results presented that circTP63 expression had no apparent change after RNase R treatment (Fig. 1F).

### Downregulation of circTP63 inhibits cell proliferation, migration and invasion in GBC

**Fig. 2:**
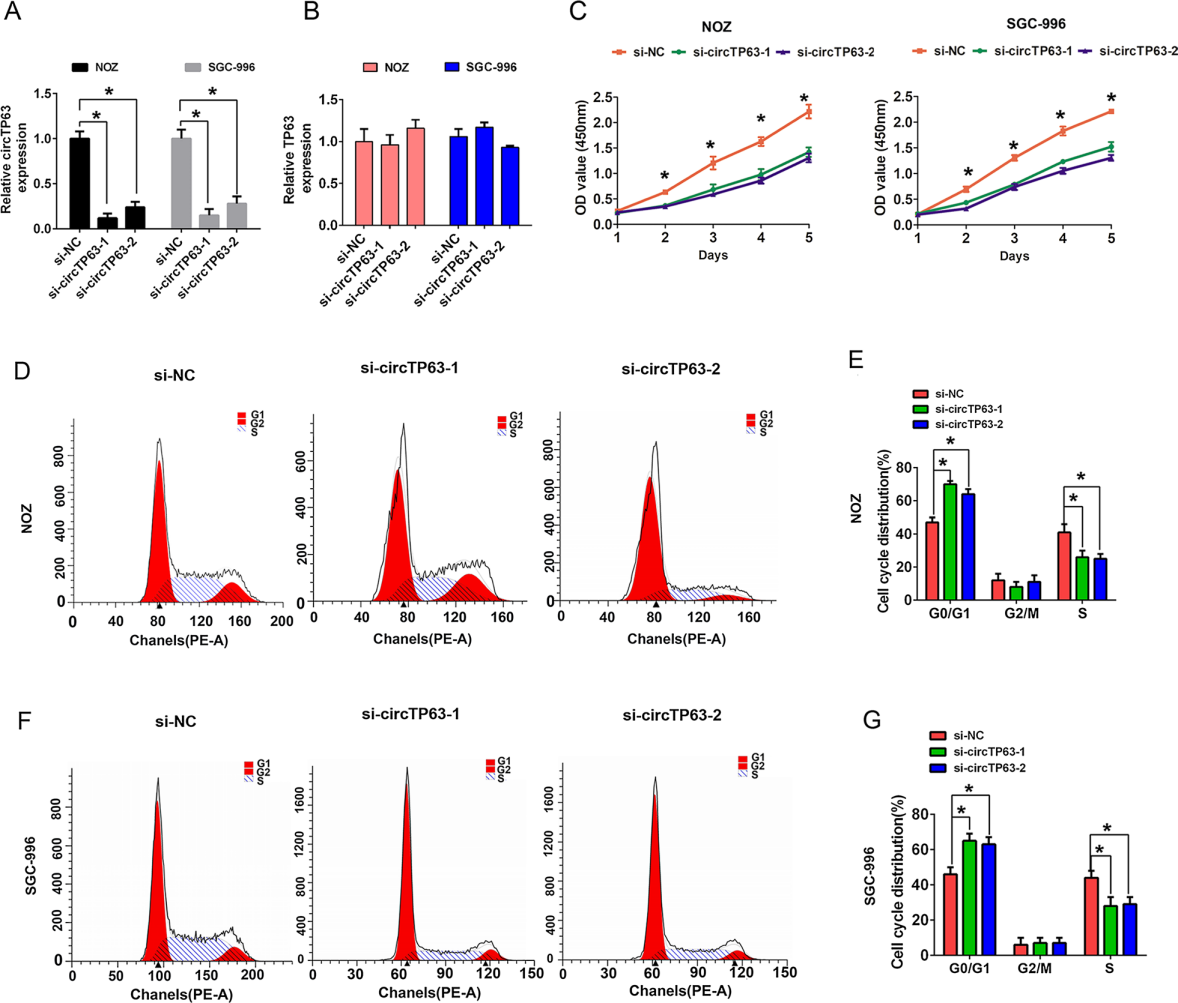
CircTP63 knockdown suppressed cell proliferation and cell cycle progress in GBC. **A** The relative expression of circTP63 was detected in NOZ and SGC-996 cells by qRT-PCR analysis after cells were transfected with si-NC, si-circTP63-1 or si-circTP63-2. **B** The relative expression of TP63 mRNA was detected in NOZ and SGC-996 cells by qRT-PCR analysis after cells were transfected with si-NC, si-circTP63-1 or si-circTP63-2. **C** The cell proliferation ability was evaluated by CCK8 assays in NOZ and SGC-996 cells after cells were transfected with si-NC, si-circTP63-1 or si-circTP63-2 at 1–5 days. **D**, **E** The cell cycle progression was analyzed by flow cytometry after cells were transfected with si-NC, si-circTP63-1 or si-circTP63-2 in NOZ cells. **F**, **G** The cell cycle progression was analyzed by flow cytometry after cells were transfected with si-NC, si-circTP63-1 or si-circTP63-2 in SGC-996 cells. Data are shown as mean  ±  SD, *P  < 0.05

**Fig. 3:**
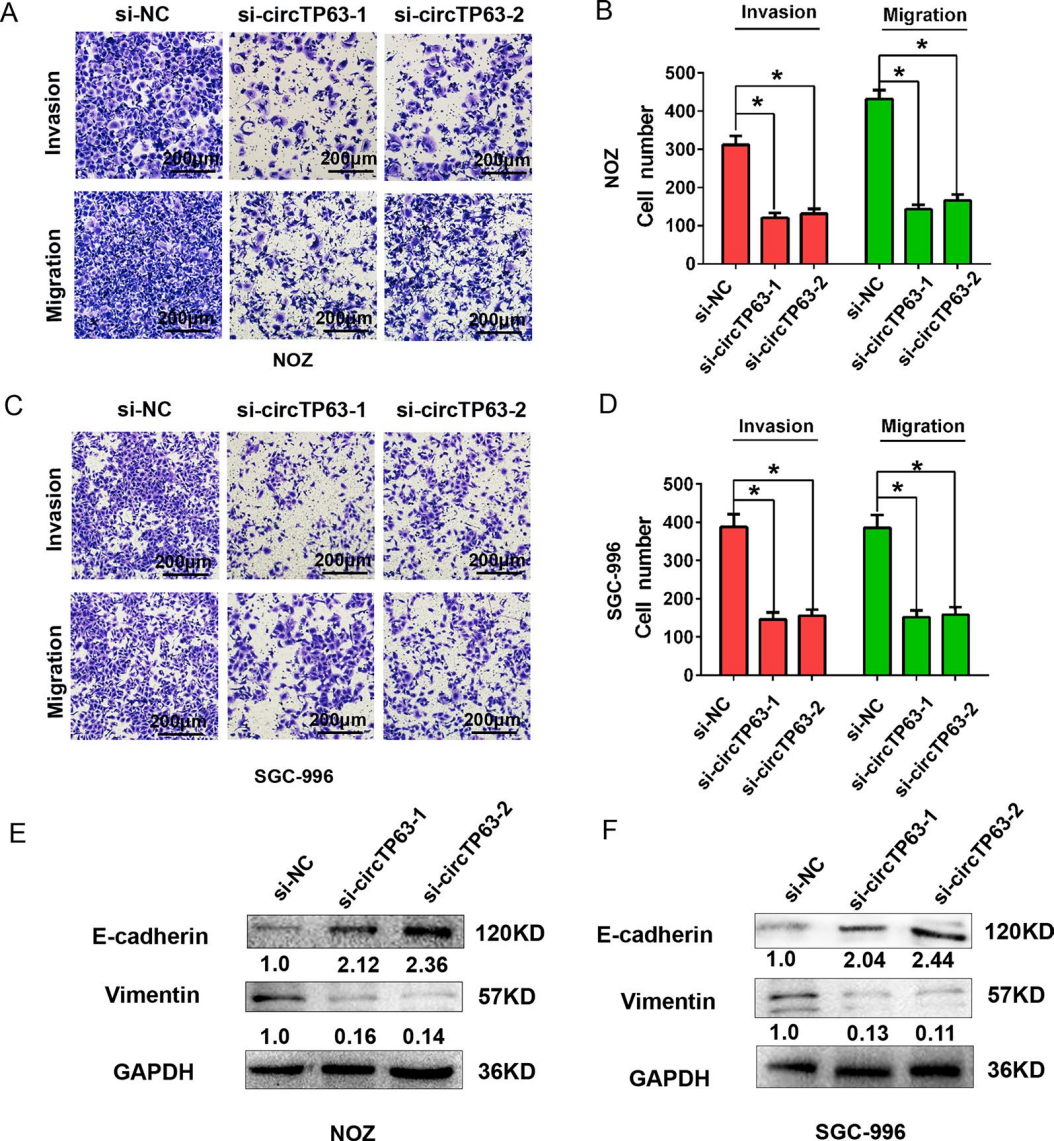
CircTP63 knockdown suppressed cell migration, invasion and cell EMT progression in GBC. **A**, **B** The cell migration and invasion abilities were analyzed by transwell assays after cells were transfected with si-NC, si-circTP63-1 or si-circTP63-2 in NOZ cells. **C**, **D** The cell migration and invasion abilities were analyzed by transwell assays after cells were transfected with si-NC, si-circTP63-1 or si-circTP63-2 in SGC-996 cells. **E**, **F** The protein expression of E-cadherin or Vimentin was analyzed by western blot analysis after cells were transfected with si-NC, si-circTP63-1 or si-circTP63-2 in NOZ or SGC-996 cells. Data are shown as mean  ±  SD, *P  < 0.05

Two specific circTP63 siRNAs were used to knock down the expression of circTP63 in GBC cells. The qRT-PCR analysis results showed that the expression level of circTP63 was markedly reduced, but the TP63 mRNA was not changed after circTP63 knockdown in NOZ and SGC-996 cells (Fig. 2A, B). Cell proliferation was assessed by CCK8 assay, and the results showed that knockdown of circTP63 suppressed cell proliferation ability compared with the control group in NOZ and SGC-996 cells (Fig. 2C). In addition, flow cytometry analysis also demonstrated that circTP63 knockdown significantly inhibited S phase cell number but increased G1 cell number compared with the control group in NOZ and SGC-996 cells (Fig. 2D–G). Moreover, transwell assay was performed to detect cell migration and invasion ability, and the results showed that the cell migration and invasion ability was impaired by circTP63 silencing compared with the control group in NOZ and SGC-996 cells (Fig. 3A–D). The epithelial marker E-cadherin expression was notably increased, while the expression of mesenchymal marker Vimentin was notably decreased after cells were transfected with si-circTP63-1 or si-circTP63-2 compared with the control group in NOZ and SGC-996 cells (Fig. 3E, F). These above evidences showed that circTP63 downregulation suppressed GBC cell growth and EMT process.

### *Downregulation of circTP63 inhibits cell growth *in vivo* in GBC*

**Fig. 4:**
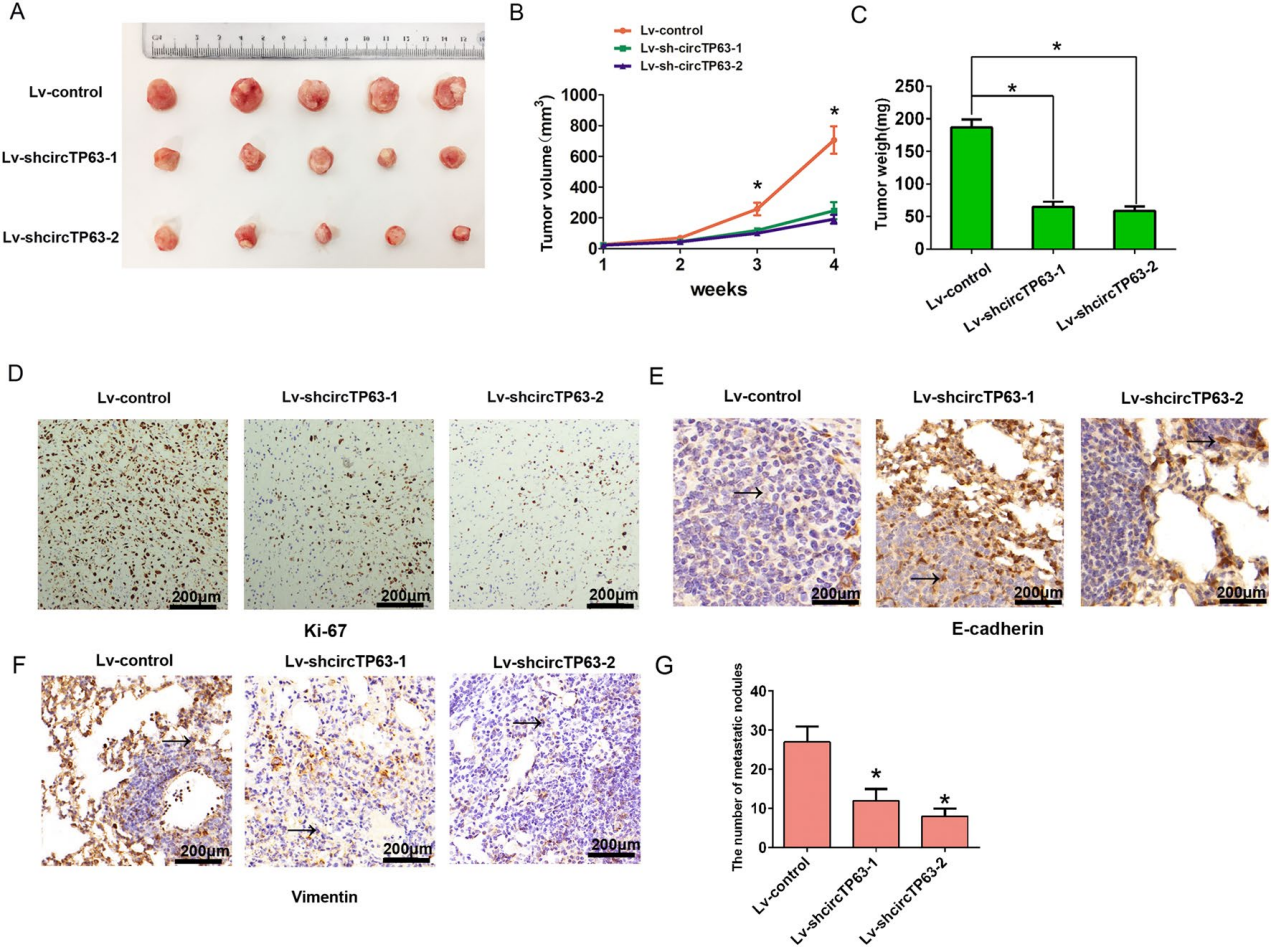
CircTP63 knockdown suppressed cell growth and EMT process in GBC in vivo. **A** Representative images of subcutaneous xenograft tumors (n  =  5 for each group). **B** Growth curves of tumor volume, which were measured every week. **C** Growth curves of tumor weight, which were measured every week. **D** The protein levels of Ki-67 were analyzed based on IHC staining. **E** The protein levels of E-cadherin were analyzed based on IHC staining. **F** The protein levels of Vimentin were analyzed based on IHC staining. **G** the metastatic nodules number was shown in lung. *P  < 0.05

To evaluate the roles of circTP63 expression in vivo, we constructed xenograft tumors in nude mice by injection with NOZ cells stably expressing sh-circTP63 or control vector by using lentiviral transduction. The results showed that the circTP63 knockdown had slower growth rate, and reduced tumor volume or weigh than those expressing in control vector (Fig. 4A–C). Immunohistochemistry staining of Ki-67 expression in xenograft tumors demonstrated that tumor tissues in sh-circTP63-1 or sh-circTP63-2 group had less Ki-67 positive cells than that in the control group (Fig. 4D). Furthermore, immunohistochemistry staining demonstrated that the epithelial marker E-cadherin expression was notably increased, while the expression of mesenchymal marker Vimentin expression was notably decreased in circTP63 knockdown group compared the control group in the metastastic nodules in lung after tail vein injection at 4 weeks, moreover, the nodules number was also reduced after knockdown of circTP63 compared with the control group (Fig. 4E, F, G). These results suggested that circTP63 knockdown could inhibit tumor growth and EMT in vivo.

### circTP63 sponges miR-217 in GBC cells

**Fig. 5:**
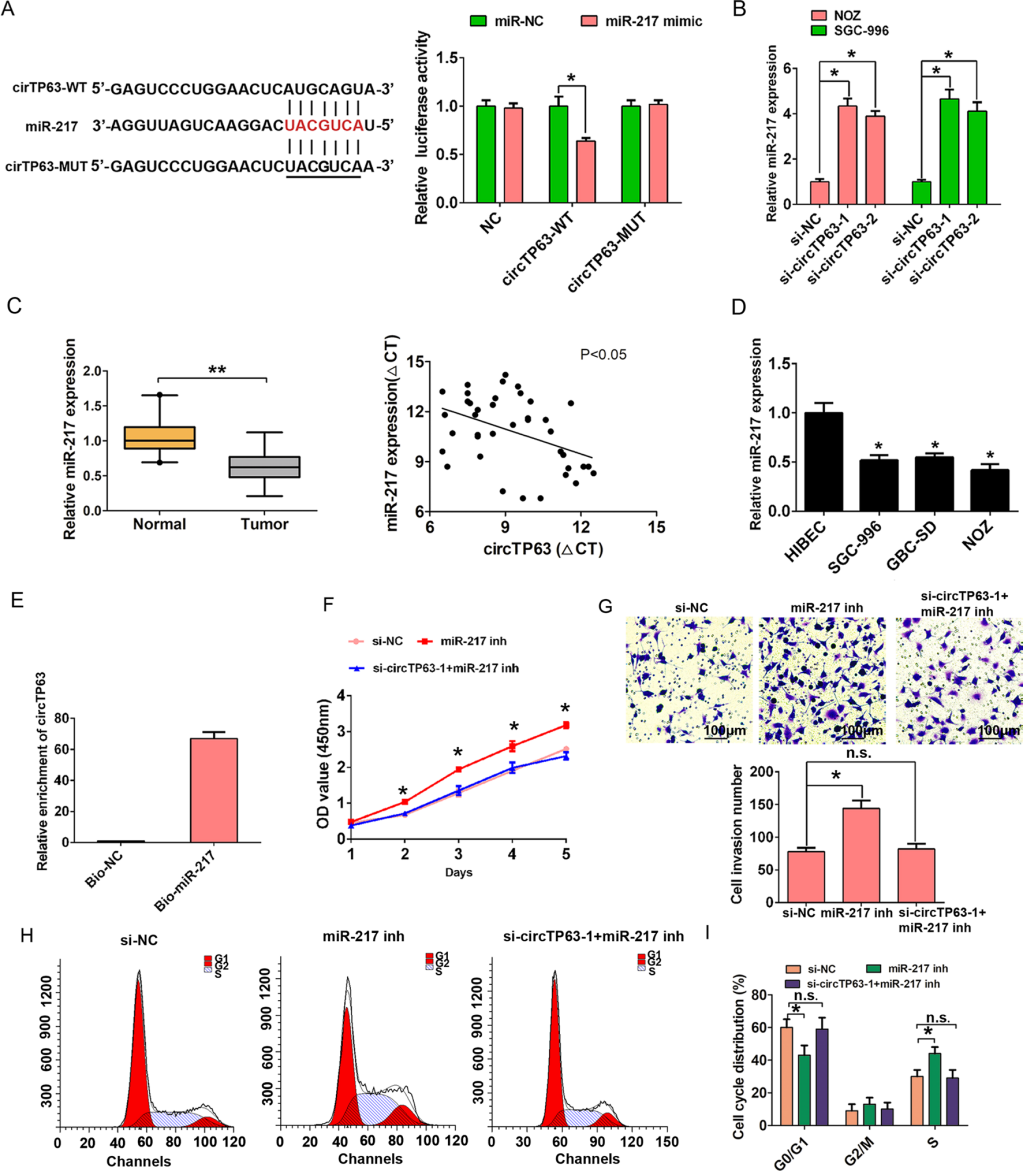
CircTP63 functions as a sponge of miR-217. **A** Schematic of the wild-type (WT) or mutant-type (MUT) circTP63 luciferase reporter vectors was constructed (left). The luciferase activities of the circTP63-WT or circTP63-MUT luciferase reporter vector in NOZ cells transfected with miR-217 mimic or miR-NC (right). **B** The relative expression of miR-217 was detected after NOZ or SGC-996 cells were transfected with si-NC, si-circTP63-1 or si-circTP63-2 by qRT-PCR analysis. **C** The relative expression of miR-217 was detected in GBC tissues and adjacent normal tissues by qRT-PCR analysis (left). Higher circTP63 expression negatively associated with lower miR-217 expression (right). **D** The relative expression of miR-217 was detected in three GBC cell lines (NOZ, SGC-996 and GBC-SD) and the human intrahepatic biliary epithelial cell line HIBEC by qRT-PCR analysis. **E** The bio-miR-217 or NC group complex was pulled down by incubating the cell lysate with streptavidin-coated magnetic beads and the circTP63 was detected by qRT-PCR. **F** The cell proliferation ability was analyzed by CCK8 assays after cells were transfected with si-NC, miR-217 inhibitor or si-circTP63-1  +  miR-217 inhibitor in NOZ cells. **G** The cell invasion ability was analyzed by transwell assays after cells were transfected with si-NC, miR-217 inhibitor or si-circTP63-1  +  miR-217 inhibitor in NOZ cells. **H**, **I** The cell cycle progression was analyzed by flow cytometry after cells were transfected with si-NC, miR-217 inhibitor or si-circTP63-1  +  miR-217 inhibitor in NOZ cells. Data are shown as mean  ±  SD, *P  <  0.05, **P  <  0.01

Recently, more studies have reported that circRNAs could sponging to miRNAs, thereby reducing the regulation of miRNAs on their target genes [15]. By performing a search for miRNAs that have complementary base pairing with circTP63 by using the online software tools circinteractome (http://circinteractome.nia.nih.gov), the results showed that miR-217 could form complementary base pairing with circTP63 (Fig. 5A, left). Luciferase assay demonstrated that miR-217 mimic repressed the luciferase activity of circTP63-WT (wild type), while miR-217 mimic had little effect on that of circTP63-MUT(mutant-type) in NOZ cells (Fig. 5A, right). We then detected the expression of miR-217 after knockdown of circTP63, the results indicated that miR-217 expression was significantly upregulated after knockdown of circTP63 in NOZ and SGC-996 cells compared with the control group (Fig. 5B). Furthermore, we also detected that miR-217 expression was significantly lower in GBC tissues compared with adjacent normal tissues by qRT-PCR analysis (Fig. 5C, left). Higher circTP63 expression was negatively correlated with lower miR-217 expression in GBC tissues by Pearson correlation analysis (Fig. 5C, right, r  =  − 0.423, P  <  0.05). The miR-217 expression was also lower expression in three GBC cell lines than that in normal HIBEC cells (Fig. 5D). In addition, we demonstrated that circTP63 was pulled down and enriched with 3′-end biotinylated miR-217 in NOZ cells compared with the control group (Fig. 5E). Functional assays were used to explore the association between circTP63 and miR-217 in NOZ cells. CCK8 assay results showed that miR-217 inhibitor significantly promoted cell proliferation ability, but was rescued by transfecting with si-circTP63-1 in NOZ cells (Fig. 5F). Transwell invasion assay results showed that miR-217 inhibitor significantly promoted cell invasion ability, but was rescued by transfecting with si-circTP63-1 in NOZ cells (Fig. 5G). Besides, flow cytometry analysis also demonstrated that miR-217 inhibitor significantly promoted S phase cell number, but was rescued by transfecting with si-circTP63-1 in NOZ cells (Fig. 5H, I). These above results showed that circTP63 affected cell proliferation and invasion by regulating miR-217 expression.

### CircTP63 sponges miR-217 and regulates EZH2 expression in GBC

**Fig. 6:**
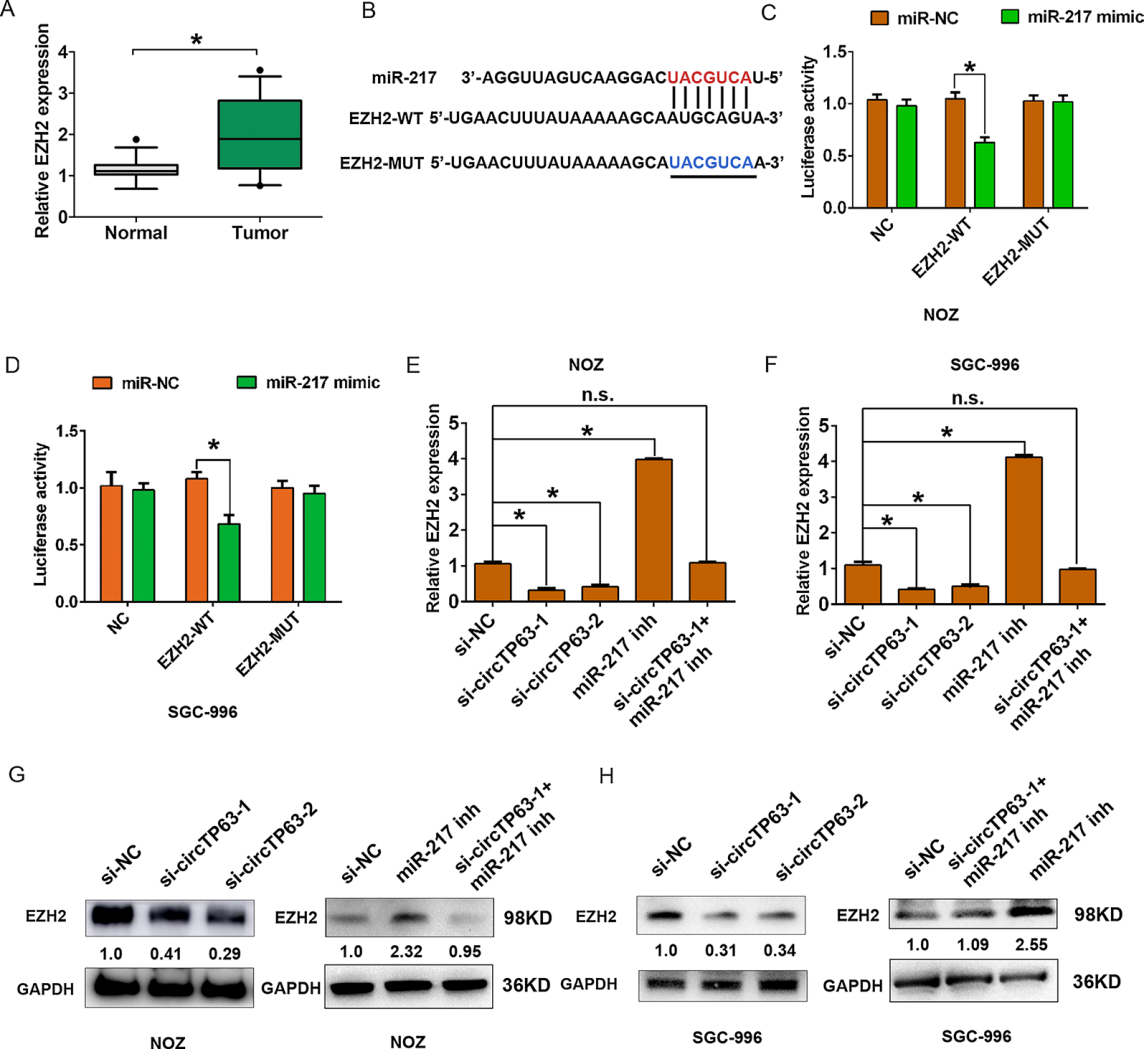
EZH2 is a downstream target of miR-217 and is indirectly regulated by circTP63. **A** The relative expression of EZH2 was detected in GBC tissues and adjacent normal tissues by qRT-PCR analysis. **B** Schematic of the wild-type (WT) or mutant-type (MUT) EZH2 luciferase reporter vectors was constructed. **C**, **D** The luciferase activities of the EZH2-WT or EZH2-MUT luciferase reporter vector in NOZ and SGC-996 cells transfected with miR-217 mimic or miR-NC. **E**, **F** The relative mRNA expression of EZH2 was detected in after NOZ or SGC-996 cells were transfected with si-NC, si-circTP63-1, si-circTP63-2, miR-217 inhibitor and si-circTP63-1  +  miR-217 inhibitor by qRT-PCR analysis. **G**, **H** The relative protein expression of EZH2 was detected in after NOZ or SGC-996 cells were transfected with si-NC, si-circTP63-1, si-circTP63-2, or si-NC, miR-217 inhibitor and si-circTP63-1  +  miR-217 inhibitor by western blot analysis, respectively. Data are shown as mean  ±  SD, *P  < 0.05

It was reported that the miR-217 could regulate the EZH2 expression in gallbladder cancer [16], we sought to explore whether circTP63 expression could affect EZH2 expression. The EZH2 expression was significantly higher in GBC tissues compared with adjacent normal tissues by qRT-PCR analysis (Fig. 6A). EZH2 was predicted as a target gene of miR-217 (Fig. 6B). Luciferase assays demonstrated that miR-217 mimic repressed the luciferase activity of EZH2-WT, while miR-217 mimic had little effect on that of EZH2-MUT in NOZ or SGC-996 cells (Fig. 6C, D). We also demonstrated that EZH2 mRNA expression was downregulated in NOZ and SGC-996 after circTP63 knockdown, but was rescued by transfecting with miR-217 inhibitor and si-circTP63-1(Fig. 6E, F). The western blot analysis showed that EZH2 protein expression was downregulated in NOZ and SGC-996 cells transfected with si-circTP63-1 and si-circTP63-2, but was rescued by transfected miR-217 inhibitor and si-circTP63-1(Fig. 6G, H). Thus, these results indicated that circTP63 sponged to miR-217 and regulated EZH2 expression in GBC. In our previous study, we demonstrated that EZH2 expression is upregulated in GBC and is a key downstream target of lncRNA MINCR, which regulates cell proliferation, cell invasive and apoptosis in GBC cells [15]. In the study, we revealed a novel regulatory pathway that circTP63 sponged miR-217 and regulated EZH2 expression in GBC.

## Discussion

Gallbladder carcinoma presents a high degree of malignancy and extremely dismal prognosis for patients. Recently, increasing studies are beginning to explore the new therapeutic methods deriving from molecular mechanisms for GBC [18]. Accumulating evidence regarding the multifunctionalities of circRNAs make them ideal for targets and markers for the prognosis, diagnosis, and developing new treatments of cancer [19]. Such as, circular RNA circ-MTO1 expression is upregulated in GBC tissues and serves as a novel potential diagnostic and prognostic biomarker for gallbladder cancer [20]. Huang et al. reported that circular RNA circERBB2 promotes gallbladder cancer proliferation in vitro and in vivo. Furthermore, circERBB2 regulates nucleolar localization of PA2G4, thereby forming a circERBB2-PA2G4-TIFIA regulatory axis to modulate ribosomal DNA transcription and GBC proliferation [21]. Our previous study reported that by RNA sequencing from GBC tissues, circFOXP1 promoted GBC progression and Warburg effect by regulating PKLR expression, suggesting a potential target for GBC treatment [22]. However, the study for circTP63 in GBC progression remains less.

In the study, our results demonstrated that circTP63 expression was upregulated in GBC tissues compared to adjacent normal tissues. We also detected that circTP63 expression is higher in GBC cell lines. Furthermore, clinical data by K–M analysis and log rank test showed that higher circTP63 expression showed a poor overall survival rate in GBC patients compared with lower circTP63 expression. These clinical results indicated that circTP63 expression could be a prognostic maker for GBC. In our previous study, microRNA and long non code RNAs have been studies for potential molecular biomarkers for GBC prognosis [15]. These clinical results indicated that a circRNA named circTP63 could be a prognostic maker for GBC.

In the previous study, CircTP63 is identified as vital regulator in several tumors. Such as, circTP63 expression is elevated in lung squamous cell carcinoma (LUSC) tissues and is correlated with larger tumor size and higher TNM stage in LUSC patients. Function studies showed circTP63 promotes cell proliferation both in vitro and in vivo by competitively binding to miR-873-3p and regulating the level of FOXM1 [12]. In breast cancer, Deng et al. found that circular RNA circTP63 enhances estrogen receptor-positive breast cancer progression and malignant behaviors through the miR-873-3p/FOXM1 axis [13]. Our results showed that circTP63 knockdown inhibited cell proliferation and cell cycle progression. Furthermore, we demonstrated that circTP63 knockdown inhibited cell migration, cell invasion ability and EMT process. These evidences indicated that circTP63 affected GBC cell growth and EMT process.

Next, we explored the underlying molecular mechanisms of circTP63 in GBC. CircRNAs were reported to exert their functions such as ‘microRNA sponge’ that competitively bound to miRNAs. In the study, we performed bioinformatic analyses to select miRNAs, which shared common binding sites with circTP63. The results showed that miR-217 shared common binding sites with circTP63. Simultaneously, we found that miR-217 reduced the luciferase activity of circTP63-WT group luciferase reporter compared to control or circTP63-MUT group. After circTP63 knockdown, the miR-217 expression was upregulated in GBC cells. In addition, we demonstrated that circTP63 was pulled down and enriched with 3′-end biotinylated miR-217 in GBC cells compared with the control group, these results indicated that circTP63 could interacted with miR-217 in GBC.

EZH2 is found to be upregulated in GBC tissues in previous study and overexpression of EZH2 is associated with invasion, metastasis, and poor progression of gallbladder adenocarcinoma [23]. Long noncoding RNA MEG3 regulates LATS2 by promoting the ubiquitination of EZH2 and inhibits proliferation and invasion in gallbladder cancer [24]. Our previous study also showed that EZH2 expression is also higher in GBC tissues and overexpression of EZH2 enhanced GBC tumor progression. Then, we showed that lncRNA MINCR/miR-26a-5p/EZH2 axis was involved in cell proliferation, cell invasive and apoptosis in GBC cells [17]. In the study, we found a novel regulating pathway that circTP63/miR-217/EZH2 affected GBC cell proliferation and invasion ability in vitro. Of course, for limitations, our result only demonstrated a regulatory role of circular RNA circTP63 in gallbladder cancer cells from a molecular level. In the further, we hope to confirm the functional role of circTP63 by knockout animal models. Besides, the expanded clinical samples are also necessary for further verification of clinical roles.

## Conclusions

In conclusion, our study firstly explored the biological significance of circTP63 in GBC. We demonstrated that circTP63 expression was significantly upregulated in GBC tissues. Higher circTP63 predicted the poor prognosis of GBC patients. CircTP63 downregulation inhibited the GBC cells proliferation and metastasis. Moreover, we found that circTP63 knockdown inhibited EZH2 expression by sponging to miR-217. Therefore, circTP63 inhibition might serve as a potential therapeutic target for GBC patients in the future.

## Data Availability

The datasets used and/or analyzed during the current study are available from the corresponding author on reasonable request.

## Abbreviations

GBC: Gallbladder cancer
OS: Overall survival
circRNAs: Circular RNAs
cDNA: Complementary DNA
qRT-PCR: Quantitative real-time PCR
SDS/PAGE: SDS-polyacrylamide gel electrophoresis
WT: Wild-type
MUT: Mutant-type
ceRNA: Competing endogenous RNA
